# A detailed review on the phytochemical profiles and anti-diabetic mechanisms of *Momordicacharantia*

**DOI:** 10.1016/j.heliyon.2022.e09253

**Published:** 2022-04-06

**Authors:** Sunday Faith Oyelere, Oluwatobi Hezekiah Ajayi, Titilayo Eunice Ayoade, George Bueno Santana Pereira, Bolaji Charles Dayo Owoyemi, Ajibola Olaoluwa Ilesanmi, Olalekan Amos Akinyemi

**Affiliations:** a Institute of Biomedical Research, Universitat de Lleida, Spain; b Department of Biology, Georgia State University, USA; c Department of Physiology, Ladoke Akintola University of Technology, Ogbomoso, Nigeria; d Department of Chemistry, Federal University of Sao Carlos, Brazil; e Department of Biological Sciences, East Tennessee State University, USA; f Department of Toxicology and Cancer Biology, University of Kentucky, USA

**Keywords:** *Momordica charantia*, Diabetes mellitus, Anti-diabetic, Phytochemical, Cucurbitaceae, Hypoglycemic, Hyperglycemia

## Abstract

Diabetes mellitus is the most well-known endocrine dilemma suffered by hundreds of million people globally, with an annual mortality of more than one million people. This high mortality rate highlights the need for in-depth study of anti-diabetic agents. This review explores the phytochemical contents and anti-diabetic mechanisms of *M. charantia* (cucurbitaceae). Studies show that *M. charantia* contains several phytochemicals that have hypoglycemic effects, thus, the plant may be effective in the treatment/management of diabetes mellitus. Also, the biochemical and physiological basis of *M. charantia* anti-diabetic actions is explained. *M. charantia* exhibits its anti-diabetic effects via the suppression of MAPKs and NF-κβin pancreatic cells, promoting glucose and fatty acids catabolism, stimulating fatty acids absorption, inducing insulin production, ameliorating insulin resistance, activating AMPK pathway, and inhibiting glucose metabolism enzymes (fructose-1,6-bisphosphate and glucose-6-phosphatase). Reviewed literature was obtained from credible sources such as PubMed, Scopus, and Web of Science.

## 1 Introduction

*Momordica charantia (M. charantia),* also known as bitter melon, karela, bitter gourd, or balsam pear, is a medicinal plant from the Cucurbitaceae family; it is predominantly cultivated in Africa, Asia, and South America [1, 2]. The name bitter guard or melon is given to it due to the fruit's bitter flavor, which becomes more pronounced as it ripens. Bitter melon is a medicinal plant with diverse beneficial effects [3], although mainly known for its anti-diabetic effects [4]. The anti-diabetic effects of *M. charantia* can be attributed to its different bioactive substances such as vicine, charantin, glycosides, karavilosides, polypeptide-p, and plant insulin [5]. These bioactive compounds belong to the broad class of phytochemicals: triterpene, protein, steroids, alkaloids, inorganic, lipid, and phenolic compounds [6, 7]. *M. charantia'*s anti-diabetic activities are reported in both type 1 and 2 diabetes mellitus. Moreover, all morphological parts of *M. charantia* demonstrated hypoglycemic activity in normal animals [8], alloxan-induced diabetic [9, 10], streptozotocin-induced diabetic model [11, 12], as well as diabetes genetic models [13]. In exploratory animal models, *M. charantia* has shown encouraging impacts in preventing diabetes mellitus and retarding the advancement of diabetic complications, including neuropathy, gastroparesis, nephropathy, waterfall, and insulin obstruction [8].

## 2 Methodology

A literature search was performed using PubMed, Scopus, and Google scholars on all original research articles as well as review articles written in English on phytochemical constituents and antidiabetics/hypoglycemic effect of *M. Charantia* within the past 25 years majorly using keywords such as ‘*Momordica Charantia’,* ‘*Momordica Charantia* + phytochemicals’, ‘*Momordica Charantia* + phytoconstituent’, ‘*Momordica Charantia* + extracts + Antidiabetics’, ‘*Momordica Charantia* + Antidiabetics’, ‘*Momordica Charantia* + hypoglycemic, ‘*Momordica Charantia* + extracts + hypoglycemic’. Figures were designed using, Corel Draw, online software.

## 3 The global burden of diabetes mellitus occurrence and mortality

Diabetes mellitus (DM), a mixture of heterogeneous problems, is usually characterized by hyperglycemia and glucose bigotry scenes resulting from the lack of insulin production, insulin resistance, or both [14]. Such complications are discernible to the absence of homeostasis in the frameworks liable for the metabolism of biomolecules [15]. DM is a significant precursor of visual impairment, kidney distress, coronary failures, stroke, and lower appendage removal [15]. It is right now a typical and genuine wellbeing concern internationally [16], and the most well-known endocrine dilemma, with approximately 690 million cases prophesied in 2045 [17]. To mitigate against this foreseen spurt in the number of diabetic patients in the near future, it is expedient to accord attention to natural products such as *M. charantia* that could be maximized in the therapy of DM.

## 4 Reported anti-diabetic activities of extracts of *M. charantia*

The anti-diabetic impacts of various extracts of *M. charantia* have been detailed in various scientific studies. Kar et al. documented the hypoglycemic effect of ethanolic sections of *M. charantia* (250 mg/kg) within 14 days of treatment in an alloxan-induced diabetic murine model [18]. Consecutive use of aqueous and ethanol extracts of *M. charantia* (200 mg/kg, orally) in alloxan- and streptozotocin- induced diabetic rats resulted in a critical reduction in plasma glucose levels after 21 days, though; the aqueous extract is found more effective [19]. The mash saponin-free methanolic concentrate of *M. charantia* has a huge antiglycemic impact on fasting and post-prandial conditions in normal, glucose-treated normal and non insulin-dependent diabetes mellitus rats [8]. *M. charantia* treatment of alloxan diabetic rats impeded cataract development, observed at 100 days in untreated diabetic rats [20]. Another study documented that, regular administration of a high dose of *M. charantia* extracts to alloxanized diabetic rats (120 mg/kg) for 2–8weeks delayed cataract progression to 140–180 days compared to 90–100 days in control rats [21]. Oral administration of aqueous extracts of *M. charantia* (400 mg/day for 15 days) to fructose-rich dietary fed rats considerably forestalled hyperglycemia and hyperinsulinemia in comparison with fructose-rich fed untreated groups [22]. Seared *M. charantia* fruits devoured as a daily food supplement influence a minor but crucial increase in glucose tolerance in diabetic animals/subjects with no expansion in serum insulin levels [23]. In another clinical investigation, a homogenized suspension of *M. charantia* given to 100 cases of moderate T2DM human subjects resulted in a significant (P < 0.001) decrease in post-prandial serum glucose (86% cases) and fasting glucose (5% cases) [8]. Welihindaa et al. reported glucose tolerance upregulation in 73% of patients with maturity-onset diabetes administered with *M. charantia* fruit juice [24].

## 5 Phytochemical contents of *Momordica Charantia*

Over the years, many phytochemicals have been isolated and identified from *M. charantia* [25]. These bioactive compounds include numerous sterols, terpenoids, phenolic compounds, proteins, peptides, amino acids, carbohydrates, fatty acids, flavonoids, vitamins, and metals.

### 5.1 Phytosterols

Phytosterols, a group of sterols, can have up to 30 carbon atoms and are present in low concentrations in plants [26]. There are >200 different known plant sterols [26] with different therapeutic activities such as anti-cholesterol [27], anticancer [28], immunomodulation [26], skin protection [29], hypocholesterolemia [30], anti-inflammatory, atherosclerotic, and antioxidant activities [31, 32, 33]. Various phytosterols identified in *M. charantia* are Daucosterol, β-sitosterol [34], Campesterol, Stigmasterol, β-sitosterol [35], β-sitosterol [36], 25ξ-isopropenylchole-5,(6)-ene-3-O-β-D-lucopyranoside [37], Δ5–avenasterol, 25,26-dihydroelasterol [38], clerosterol, 5α-stigmasta-7-en-3β-ol [39], β-sitosterol, Stigmasterol, and Diosgenin [40].

### 5.2 Terpenoids

Terpenoids are the largest and most far-reaching class of secondary metabolites, predominantly in plants and lower spineless creatures [41]. Their biological activities include anticancer, anti-inflammatory [42], plant growth promotion [43] and reduction of cardiovascular disease. The predominant terpenoids found in *M. charantia* are cucurbitane-type terpenoids which include, 3-[(5β,19-epoxy-19,25-dimethoxycucurbita-6,23-dien-3-yl)oxy]-3-oxopropanoic acid, (3-[(5β,19-epoxy-19,25-dimethoxycucurbita-6,23-dien-3-yl)-2-oxoacetic acid, 3-[(5-formyl-7β-methoxy-7,23S-dimethoxycucurbita-5,23-dien3-yl)oxy]-3-oxopropanoic acid, 3-[(5-formyl-7β-hydroxy-25-methoxycucurbita-5,23-dien-3-yl)-oxy]-3-oxopropanoic acid, 3-[(5-formyl-7β,25- dihydroxymethoxycucurbita-5,23-dien-3-yl)- oxy]-3- oxopropanoic acid, and 3-[(25-O-methylkaravilagenin D-3- yl)oxy]-2-oxoacetic acid [44]. Other active terpenoids identified in *M. charantia* are charantin A and B, 3b,7b,25-trihydroxycucurbita-5,(23E)- dien-19-al, 28-O-β-D-xylopyranosyl, (1→3)-β-D-xylopyranosyl, 3*β*,7*β*-dihydroxy-25-methoxycucurbita-5,23- diene-19-al [45], charantagenins D and E [46], kuguaosides A, B, C and D, charantoside A, momordicosides I, F1, F2, K, L and U, goyaglycosides-b, goyaglycosides-d, 3-O-β-D-allopyranoside, 25-hydroxy-5β,19-epoxycucurbita-6,23-dien-19-on-3β-ol, 7β,25-dihydroxycucurbita-5,23(E)-dien-19-al, 3-O-β-D-glucopyranoside [47], phytol [48] Kuguacin B, J, L, M, P and S [49], 5β,19-epoxy-25- methoxy-cucurbita-6,23-diene-3b,19-diol [38], (1→4)-α-L-rhamnopyranosyl, (1→2)-[α-L-rhamnopyranosyl, 3-O-β-D-glucopyranosyl, (1→2)-β-D-glucopyranosiduronic acid, (1→3)]-β-D-fucopyranosylgypsogenin, (1→2)-[α-L-rhamnopyranosyl, (1→3)]-β-D-fucopyranosylgypsogenin, 28-O-β-D-xylopyranosyl, (1→4)-α-L-rhamnopyranosyl, (1→2)-β-D-glucopyranosiduronic acid, 3-O-β-D-glucopyranosyl, [50], 5β,19-epoxycucurbitane triterpenoids [51], karavilagenin F, karaviloside XII and XIII, momordicine I, II, VI, VII and VIII [52].

### 5.3 Fatty acids

Organic compounds with saturated or unsaturated carbonic chain terminated by a carboxyl group (-COOH) are generally known as fatty acids [53, 54]. Among other roles, plant fatty acids can forestall or decrease the danger of creating cardiovascular sicknesses [55]. Their anti-bacteria [56] and anti-fungal [57] properties have also been reported. The various fatty acids found in *M. charantia* include palmitic [58, 59, 60, 61, 62]; myristic [58, 61, 63], pentadecanoic [58, 61, 63]; arachidic [58, 59, 60, 62, 63]; palmitoleic acids [58, 61, 63], stearic [35, 60, 62, 64], oleic [58, 59, 60, 62, 63], α-linolenic [58, 61, 62, 63], linoleic [58, 59, 60, 63], capric [59], lauric [59, 61, 63], docosanoic [61, 63], heneicosanoic [61, 62, 63], nonadecanoic [61, 63], decanoic [61, 63], tridecanoic [61, 62, 63], gadoleic acids [60], α-eleostearic [35, 60], heptadecanoic [61], tetracosanoic acids [61], behenic and lignoceric acids [62].

### 5.4 Phenolic compounds

Phenolics are auxiliary metabolites found in plants with benzene-like structure. They exist as coumarins, flavonoids, lignins, lignans, ordinary phenols, phenolic acids, and tannins [65]. The pharmacological effects of phenols include antioxidant, anti-microbial, anti-HIV-1, and anticancer activities [66, 67, 68, 69]. Various phenolic compounds isolated from *M. charantia* include gallic, kaempferol, chlorogenic, caffeic acid, catechin, rutin, quercetin [70], ellagic acids [71], epicatechin [71, 72], quercitrin, isoquercitrin, [71], ferulic acids, protocatechuic [72, 73, 74], tannic [72], vanillic, p-coumaric, p-hydroxylbenzoic, [72, 74], epigallocatechin, gallocatechingallate [72], myricetin, syringic [73, 74], apigenin, apigenin-7-O–glycoside, 3- coumaric, 4- coumaric acids, luteolin, luteolin-7-O-glycoside, naringenin-7-O-glycoside [73], biochanin a, gentisic, hesperidin, homogentisic acids, naringenin, naringin, β-resorcylic, salicylic, tcinnamic and veratric acids [74].

### 5.5 Amino acids

The fruits of *M. charantia* have been shown to possess certain amino acids. These amino acids are both essential and non-essential amino acids; they include alanine, aspartic acid, butyric acid, g-amino, glutamic acid, isoleucine, leucine, luteolin, methionine, phenylalanine, pipecolic acid, serine, threonine, and valine [75]. All amino acids have a general molecular structure contains a chiral center and two functional groups – amino and carboxyl groups.

### 5.6 Vitamins

The presence of specific vitamins, which include vitamin A, vitamin E, vitamin C, vitamin B_12,_ and folic acids, have been confirmed in small quantities in the dried leave of *M. charantia.* Constrastively*,* vitamin B_3_, vitamin B_6_, vitamin D, and vitamin K are found in trace amounts in the plant's methanol and pet-ether leaf extract [76].

### 5.7 Peptides and proteins

Proteins, a class of large biomolecule, have diverse biological roles in living organisms. From various morphological parts of *M. charantia*, a variety of peptides and proteins have been discovered and extracted. Various proteins isolated from *M. charantia* are highlighted below.

#### 5.7.1 Ribosome inactivating proteins (RIPS)

Ribosome inactivating proteins (RIPs), a class of proteins, have drawn the attention of numerous specialists by virtue of their conceivably exploitable bioactivities. Ribosome-inactivating proteins are toxic *N*-glycosidases that depurinate eukaryotic and prokaryotic rRNAs, thereby arresting protein synthesis during translation [77]. RIPs are classified as type I or type II based on the number of subunits they contain. Type I RIPs isolated from *M. charantia* are single-chained. RIPs isolated and characterized from *M. charantia* are α-, β-, γ-, δ- and ε-momorcharin, momordica anti-HIV protein (MAP30), momordica charantia lectin, momordin, and trichosanthin. Various pharmacological activities of RIPs include anticancer, anti-microbial, anti-tumor, DNase-like, immunosuppressive, phospholipase, RNA N-glycosidase and superoxide dismutase, activities [78, 79, 80, 81].

#### 5.7.2 Polypeptide-P

Polypeptide-P is a hypoglycemic glycoprotein peptide. It is derived from *M. charantia's* fruit, seeds, and tissues [82]. Two types of polypeptide-P with molecular weights of approximately 11 kD (166 amino acids) and 3.4 kD have been isolated from *M. charantia* [83]. It is crucial in cell recognition and adhesion reactions and has also been isolated from bitter melon [84].

#### 5.7.3 Inhibitory proteins

Inhibitory proteins such as elastase inhibitors [85], α-glucosidase inhibitor [86], guanylatecyclase inhibitors [87], trypsin inhibitors (MC-I, -II and -III) [88], HIV inhibitory proteins like MRK29 (28.6 kDa) [89], MAP30 (30 kDa) and lectin [82] are isolated from *M. charantia*.

#### 5.7.4 P-insulin

P-insulin, a phytoconstituent of *M. charantia,* is supposed to be a polypeptide hypoglycemic substance with a molecular weight of ∼11 kDa and comprises 166 amino acids [83]. P- insulin is found in bitter melon fruits, seeds, and several tissue cultures [3].

#### 5.7.5 Other proteins

Apart from the specific proteins mentioned above, other proteins and peptides documented in *M. charantia* are peroxidase (43 kDa), momordica cyclic peptides [90], antifungal protein, cysteine knot peptides, MCha-Pr, and RNase MC2 (weight, 14 kDa) [91].

### 5.8 Polysaccharides

Polysaccharides rank among the essential bioactive constituents of *Momordica charantia*. The polysaccharides contents of *M. charantia* may be influenced by different conditions [92]. These polysaccharides are composed of different saccharide units, including arabinose, galactose, glucose, mannose, and rhamnose, and are thus classified as heteropolysaccharides [93]. The major polysaccharides isolated from *M. charantia* are shown in Table 1.

**Table 1:** List of polysaccharides isolated from *Momordica charantia*, their characteristics, and biological functions.

Types of polysaccharides	Composition	Ratio of composition	Molecular weight	Biological functions	References
Acidic and branched heteropolysaccharide	galacturonic acid, mannose, rhamnose, galactose, glucose, xylose and arabinose	0.01: 0.15: 0.02: 0.38: 0.31: 0.05: 0.09	92 kDa	antioxidant, α-amylase inhibition and angiotensin-converting enzyme inhibition	[94]
Pectic polysaccharide	1,4,5-tri-O-acetyl-2,3,6-tri-O-methyl-D-galactitol, 1,2,4,5-tetra-O-acetyl-3,6-di-O-methyl-D-galactitol and 1,5-di-O-acetyl-2,3,4,6-tetra-O-methyl-D-galactitol	3:1:1	20 kDa	Undefined	[95]
Water-soluble polysaccharides	Arabinose, xylose, galactose and rhamnose	1.00: 1.12: 4.07: 1.79	1.15 × 10^6^ Da	hypoglycemic effect	[96]

Majorly, *M. charantia* polysaccharides improve cell death, hyperlipidemia, inflammation and oxidative imbalance during myocardial infarction by hindering the nuclear factor kappa-light-chain-enhancer of activated B cells (NF-κB) flagging pathway [97]. *M. charantia* polysaccharides additionally could improve overall volatile fatty acids generation, regulate the rumen fermentation pathway and impact the quantity of cellulolytic bacteria populace [98].

## 6 Mechanisms of anti-diabetic effect of *M. charantia*

Several scientists have researched the hypoglycemic and antiglycemic impacts of the various concentrates and compounds of *M. charantia* in human and animal models [8, 83]. *M. charantia* and its various concentrates and extracts applied their hypoglycemic impacts through various pharmacological, physiological, and biochemical modes [99, 100]. The reported modes of *M. charantia* anti-diabetic exercises include hypoglycemic activity [39, 94], incitement of glucose to the peripheral and skeletal muscles [95], restriction of intestinal glucose take-up [96, 101], hindrance of adipocyte differentiation [102], concealment of main gluconeogenic enzymes [103], incitement of the main biocatalyst of glycolytic pathway [104], and safeguarding of islet β cells and their capacities [105].

**Figure 1:**
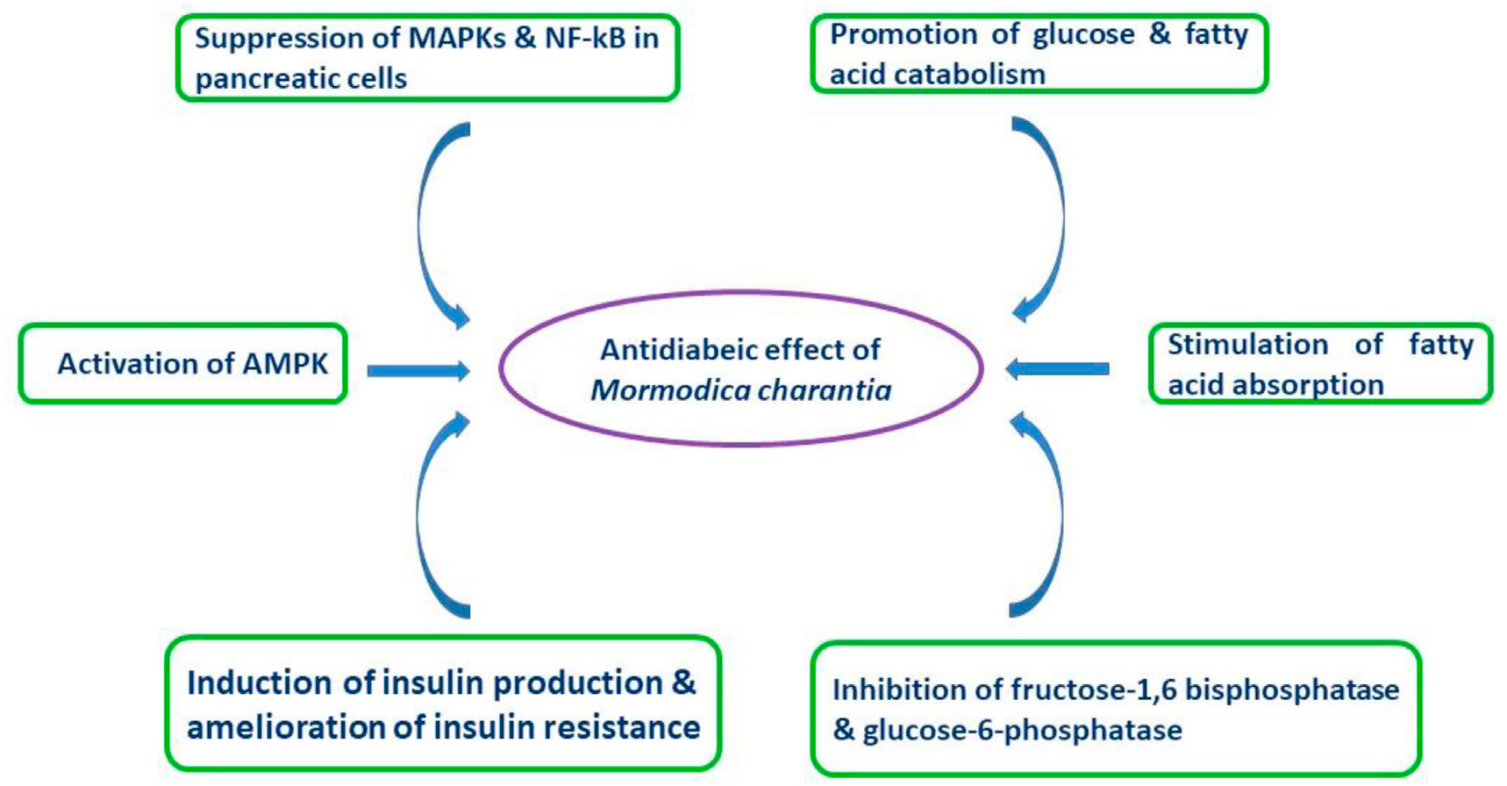
Mechanisms of the anti-diabetic effects of *Momordica charantia.*

In this review, -we explicitly show that *M. charantia* exhibits its anti-diabetic effects through the suppression of mitogen-activated protein kinases (MAPKs) and NF-κβ in pancreatic cells, promotion of glucose and fatty acids catabolism, stimulation of fatty acids absorption, induction of insulin production, amelioration of insulin resistance, activation of AMP –-activated protein kinase (AMPK), and inhibition of glucose metabolism enzymes (fructose-1,6-bisphosphate and glucose-6-phosphatase) (Figure 1).

### 6.1 Suppression of MAPKs and NF-кB in pancreatic Β-cells

**Figure 2:**
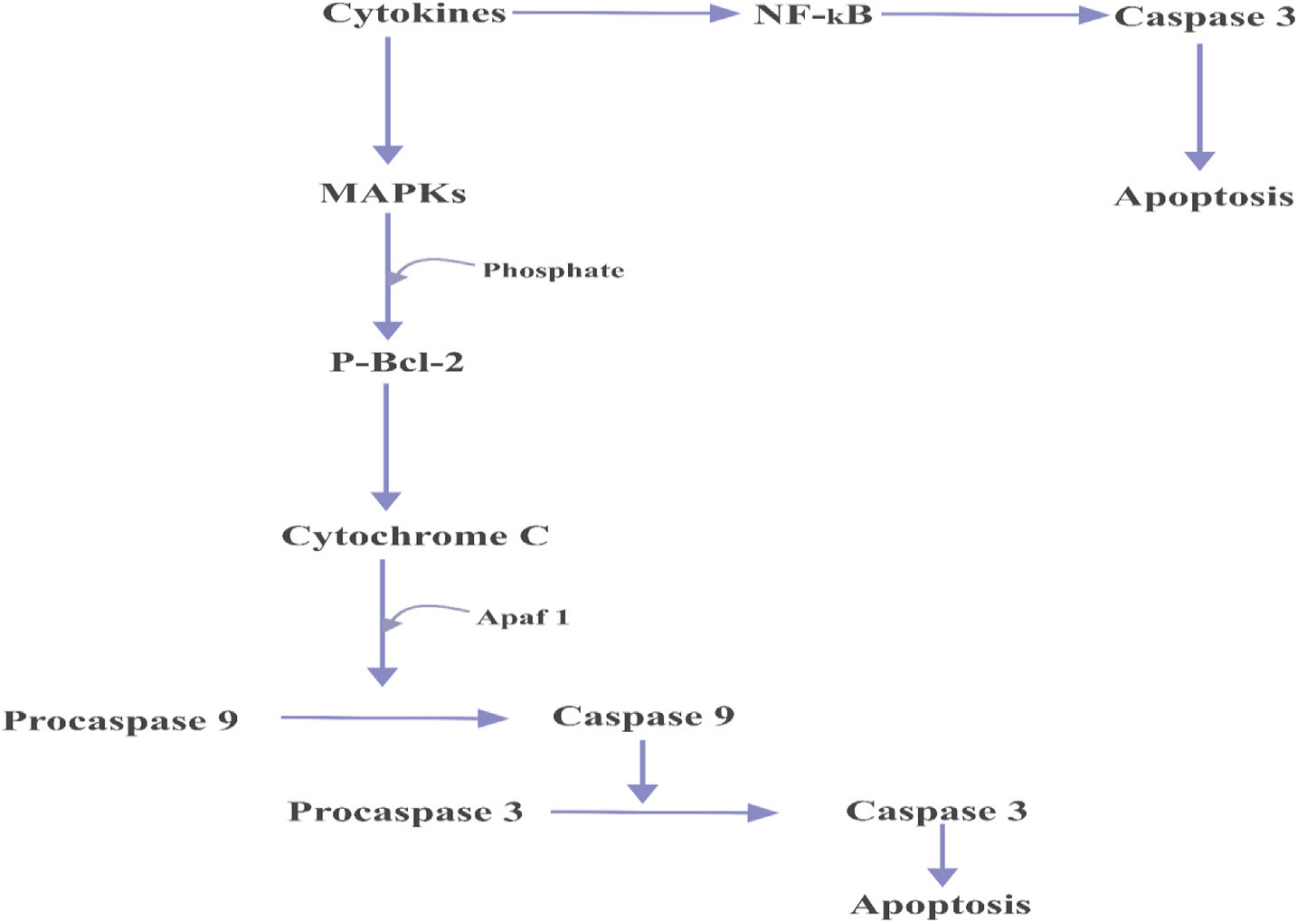
Mechanisms of pancreatic β-cells death. *Cytokines trigger apoptosis of pancreatic β-cells in two ways. (1) Cytokines (-IL-1β, IFN-γ, and TNF-α) activates MAPKs (SAPK/JNKs, p38 MAPK, and p44/42 MAPK or ERKs); the activated MAPKs phosphorylate BCl-2; the phosphorylated Bcl-2 activates cytochrome C; the activated cytochrome C recruits Apaf 1 and together converts procaspase 9 to caspase 9; caspase 9 converts procaspase 3 to caspase 3, leading to cell death. (2) Alternatively, activation of NF-κB by cytokines leads to the release of caspase 3; culminating in cell death.*

Cellular death of pancreatic β-cells is a key event in the pathogenesis of type 1 and type 2 diabetes [106]. The apoptosis of the β-cell is a systemic process triggered by cytokines family- interleukin-1β (IL-1β), interferon-gamma (IFN-γ), and tumor necrotic factor-alpha (TNF-α). These cytokines actuates several MAPKs such as stress-activated protein kinase/c-Jun N-terminal kinases (SAPK/JNKs), p38 MAPK, and p44/42 MAPK or extracellular-regulated protein kinases (ERKs), and NF-κB [107], thus leading to the pancreatic β-cells death (Figure 2) [108]. IL-1β triggers cell death by activating SAPK/JNK, p38, and p44/42 MAPKs [107]. SAPK/JNKs phosphorylates Bcl-2 which culminated in the release of mitochondrial cytochrome C [109]; p38 triggers apoptotic death of pancreatic β-cells in a similar manner [110]. SAPK/JNK is also triggered via the synergistic action of IFN-γ and TNF-α [111]. Cytokines can also promote cell death via the activation of NF-κB; NF-κB activation leads to the actuation of caspase-3 activity [112].

Kim and Kim [113] detailed that *M. charantia* aqueous ethanol can inhibit the cytokine-induced pancreatic β-cells death by stifling the actuation of mitogen-activated protein kinases (MAPKs), including stress-activated protein kinase/c-Jun N-terminal kinase (SAPK/JNK), p38, and p44/42 MAPK, MEK 1/2 and the activity of NF-κB in a pancreatic β-cells animal model (SV40 T-transformed insulinoma MIN6N8 cells derived from nonobese diabetic mice).

### 6.2 Promotion of glucose and fatty acids catabolism and fatty acid absorption

**Figure 3:**
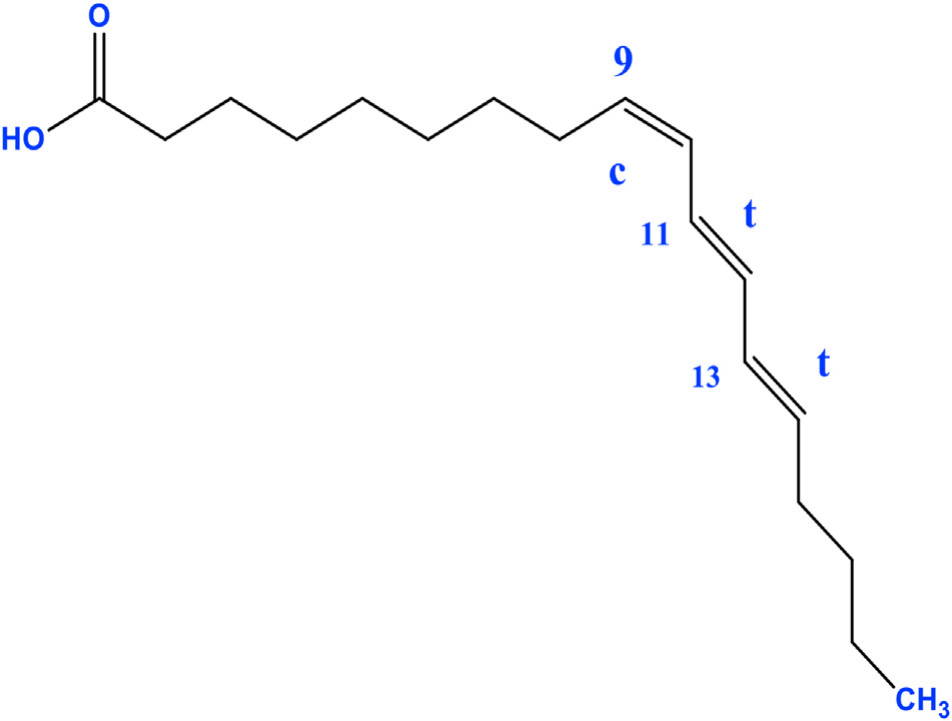
Structure of 9c,11t,13t-CLN.

One study revealed that the *M. charantia* seeds improve the serum and liver lipid profiles and serum glucose levels by inducing the expression of the peroxisome proliferator- activated receptor gamma (PPAR-γ) gene in the adipose tissue [105]. 9c,11t,13t-CLN is the phytochemical compound involved in the activation of PPAR-γ in *M. charantia* (Figure 3) [114].

**Figure 4:**
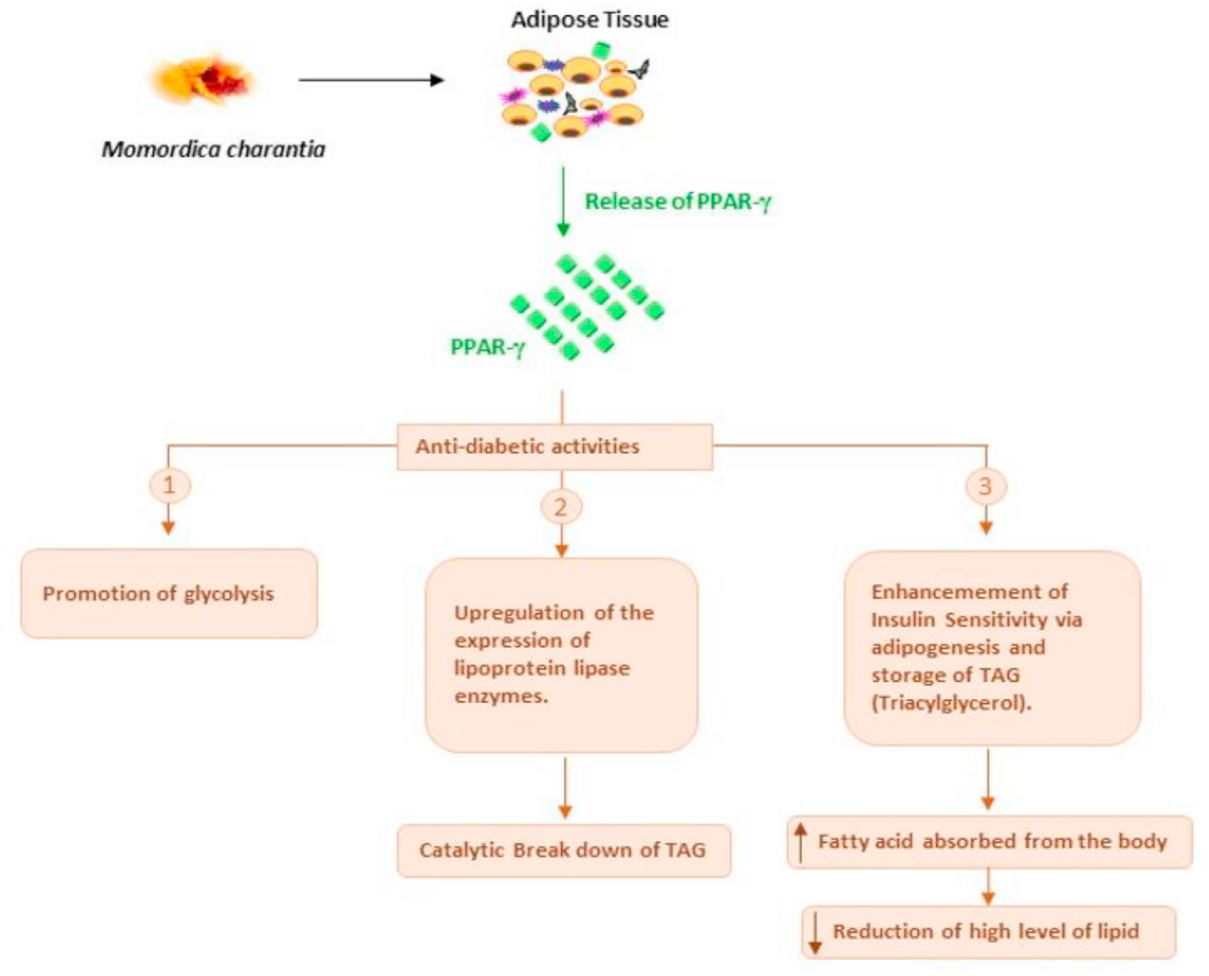
*M. charantia* improves serum and hepatic lipid profiles and blood sugar levels. *M. charantia* induces the release of PPAR-γ from the adipose tissue and the released PPAR-γ exhibits anti-diabetic action via three means: (1) by increasing the rates of glycolysis (2) degradation of TAG by increasing the expression of lipoprotein lipase enzymes (3) enhancement of insulin sensitivity by stimulating adipogenesis and increasing the storage of TAG; this leads to increased fatty acid absorption and reduction in lipid level.

PPAR-γ is a member of PPARs, a subfamily of ligand-activated transcription factors of the nuclear hormone receptors superfamily [115]. PPARs, generally a critical factor in the regulation of the many genes, are involved in coordinating several cellular and metabolic processes such as metabolism of glucose, lipoprotein and triglyceride, energy homeostasis, de novo lipogenesis, uptake, storage, oxidation, and transport of fatty acid, etc. [116, 117, 118, 119, 120]. *M. charantia* seed ameliorates hyperlipidemia and hyperglycemia by acting as a PPAR-γ ligand activator, which stimulates the expression of genes involved in lipid catabolism and glucose utilization (Figure 4) [121]. The stimulation of PPAR-γ has been proven to reduce plasma triglyceride and free fatty acids levels by promoting their breakdown through the induction of lipoprotein lipase [122]. Furthermore, PPAR-γ stimulates cellular differentiation, enhances lipid storage, and regulates insulin activities in the adipose tissue [123]. Activators of PPAR-γ also enhance insulin sensitivity via adipogenesis stimulation and post-prandial fatty acid/triacylglyceride storage within the adipocytes [124].

### 6.3 Induction of insulin production and amelioration of insulin resistance

**Figure 5:**
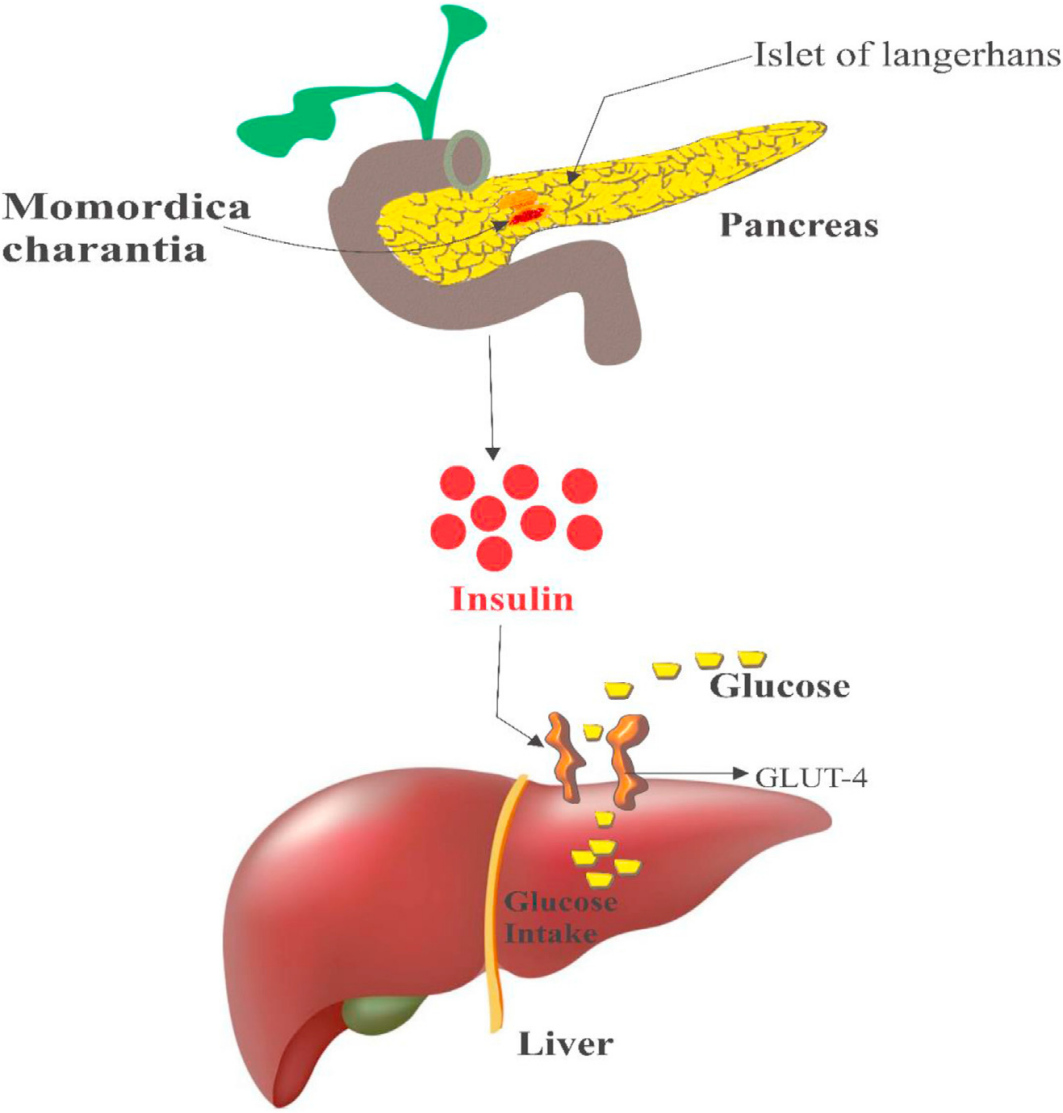
Induction of insulin discharge from β-cells of islets of Langerhans. *M. charantia* induces the secretion of insulin from the β-cell of the islet of Langerhans in the pancreas. The released insulin recruits GLUT-4 transporters which allow the absorption of glucose into the liver.

Jeewathayaparan et al. [125] exhibited that oral administration of *M. charantia* could prompt insulin emission from endocrine pancreatic β cells; this result was later corroborated by Ahmed et al. [126], who explored the impact of the day to day oral administration of *M. charantia* natural product juice on the action of α, β and δ cells in the pancreas of STZ-initiated diabetic rodents. Administration of *M. charantia* alcohol concentration to alloxan-induced diabetic rats shows a strong hypoglycemic effects and significantly improved the islets of Langerhans [127]. Other studies showed that *M. charantia* could stimulate the emission of insulin from the endocrine pancreas and elicit glucose absorption in the liver (Figure 5) [101]. We proposed a mechanism by which the aforementioned effects are achieved - the recruitment of GLUT-4 transporter (Figure 5).

### 6.4 Activation of AMP-activated protein kinase alpha

**Figure 6:**
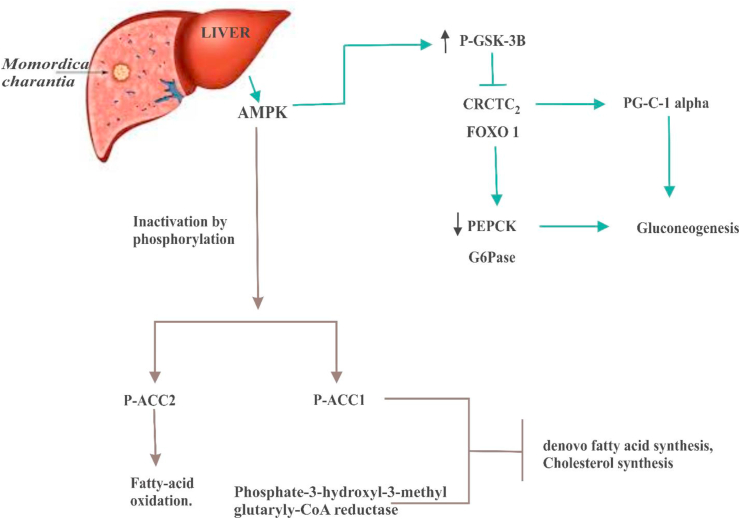
*M. charantia* inhibits gluconeogenesis, fatty acid synthesis, and cholesterol synthesis in the liver via the activation of AMPK. AMPK inhibits gluconeogenesis by suppressing the action of CRCTC2 and FOXO1 (genes that are critical in the activation of gluconeogenesis) either directly or indirectly (by increasing the synthesis of P-GSK-3β). The suppression of CRCTC2 and FOXO1 can promote the synthesis of PG-C1α or decrease the action of phosphoenolpyruvate carboxykinase (PEPCK) and glucose-6-phosphatase (G6Pase). AMPK also inactivates acetyl-CoA carboxylase 1 (ACC1) and 3-hydroxyl-3-methylglutaryl CoA reductase leading to the inhibition of de novo synthesis of fatty acid and cholesterol synthesis. ACC2 is also phosphorylated by AMPK, resulting in increased fatty acid oxidation.

**Figure 7:**
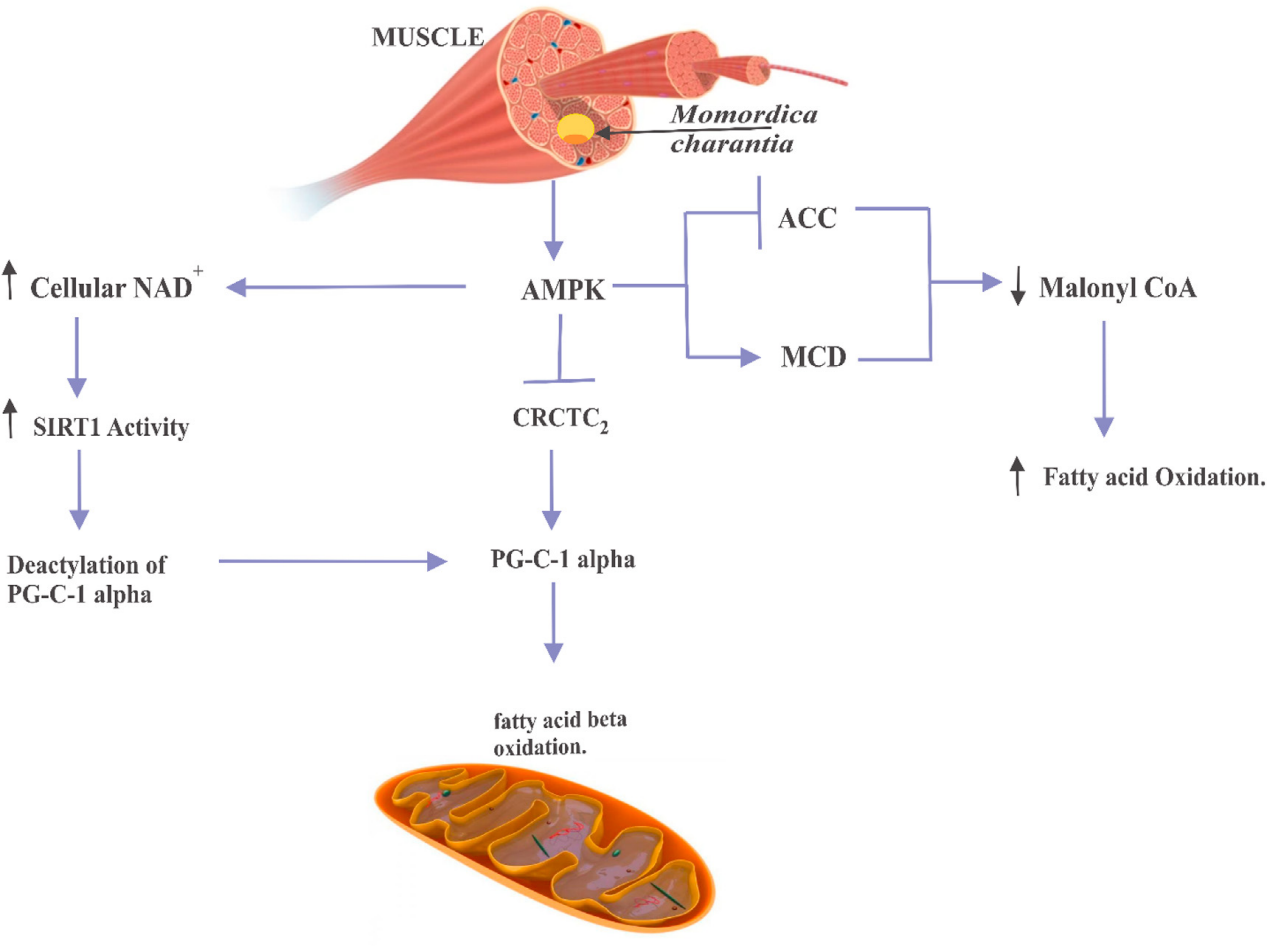
*M. charantia* upregulates fatty acid oxidation in the muscle via the activation of AMPK. *M. charantia* induces the activity of AMPK in the muscle. AMPK increases the cellular level of NAD + which further increases the activity of Sirtuin 1 (SIRT1) leading to the activation of Peroxisome proliferator-activated receptor gamma coactivator 1-alpha (PGC-1α) via deacetylation and the activated PGC-1α promotes the catabolism of fatty acid in the mitochondria. Suppression of CRCTC2 by AMPK also promotes the activation of PGC-1α, leading to the catabolism of fatty acid in the mitochondria; AMPK increases fatty acid catabolism by decreasing the level of malonyl CoA via a coordinated inhibition of ACC and activation of malonyl-CoA decarboxylase (MCD).

*M. charantia* fruits have likewise indicated the capacity to upgrade cells' glucose take-up, advance insulin discharge, and potentiate insulin's impact. Bitter melon's bioactive content enacts a protein called AMPK (AMP-activated protein kinase α), which is notable for controlling energy given foods digestion and empowering forms of glucose take-up, which are impeded in diabetes patients [128]. The mechanisms of anti-diabetes activities of AMPK are well characterized in the liver and the muscle tissues [129]. In the liver AMPK inhibits gluconeogenesis by suppressing the synthesis of key genes such as CREB-regulated transcription co-activator 2 (CRTC2) and forkhead Box O1 (FOXO) [130]. The actions of AMPK in the liver also leads to inhibition of de novo fatty acid synthesis and cholesterol synthesis as well as activation of fatty acid catabolism (Figure 6) [131]. *M. charantia* can also induce activation of AMPK in the muscle tissue, resulting primarily into an increment of fatty acid oxidation in the mitochondria and cytoplasm (Figure 7) [132].

### 6.5 Inhibition of fructose-1,6-bisphosphatase, and glucose-6-phosphatase

**Figure 8:**
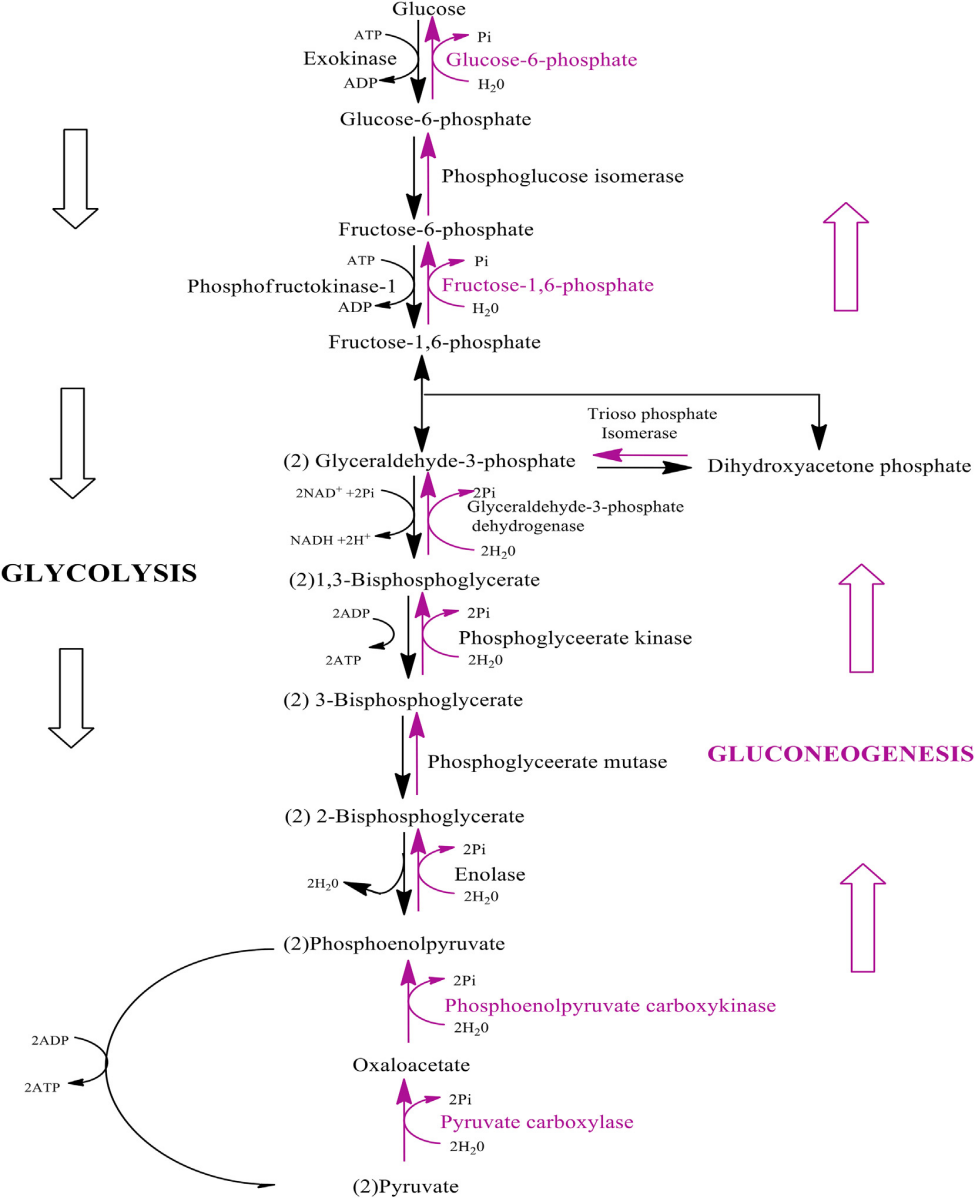
Gluconeogenesis and glycolysis pathway. *M. charantia* suppresses the activities of fructose-1,6-bisphosphate and glucose-6-phosphatase.

Fructose-1,6-bisphosphatase and glucose-6-phosphatase activities are repressed by aqueous and alcoholic concentrates of *M. charantia* [5]. Fructose-1,6-bisphosphatase catalyzes the hydrolysis of fructose-1,6-bisphosphate to fructose 6-phosphate (Figure 8) [133]. This reaction occurs in both gluconeogenesis and the Calvin cycle [134]. Fructose-1,6-bisphosphatase is a rate-limiting enzyme in gluconeogenesis and a key target for T2DM treatment due to the well-known involvement of abnormal endogenous glucose production in the disease's hyperglycemia [135]. Inhibition of fructose-1,6-bisphosphate has been proposed as a potential treatment for T2DM [136, 137]. Gluconeogenesis is a major contributor to surfeit glucose in this disease. Reducing its excess would help alleviate the pathology linked to elevated glucose concentrations in the blood and tissues. Inhibiting fructose 1,6-bisphosphatase only affect gluconeogenesis but not glycolysis [138, 139, 140, 141].

Glucose-6-phosphatase (also known as G-6-Pase), which is primarily found in the liver [142], catalyzes the final stage for both glycogenolysis and gluconeogenesis by changing glucose-6- phosphate to inorganic phosphate and glucose (Figure 8) [143, 144], making it an important regulator of blood glucose homeostasis [145]. The enzyme activity is several times higher in diabetic animals and, most likely, in diabetic humans, implying that it may be involved in the increased hepatic glucose production seen in T2DM [146]. Further, in the diabetic condition, the presence of both G-6-Pase (and glucokinase) in pancreatic -cells might result in higher glucose cycling, which can compromise glucose sensing and insulin secretion. Previous studies have shown an association of attenuated insulin production with higher glucose-6-phosphatase activity as well as glucose cycling in T2DM animal models [147, 148]. Therefore, *M. charantia* – a compound that inhibits the glucose-6-phosphatase enzyme complex – could be maximized in the treatment of T2DM.

## 7 Future perspective

Approval of any therapeutic substance and its application in pharmaceutical industry for human use is subjected to the success of the substance in clinical trial studies. While *M. charantia* and its extracts are widely regarded traditionally as a potent anti-diabetic concoction, up to date, there is scarcity of clinical trial studies on the anti-diabetic effects of the plant [8]; hence, the global acceptance of this purported “potent” antidiabetic plant in the treatment of diabetes mellitus is retarded. Unfortunately, the currently approved antidiabetic therapy has not shown maximum success, therefore more clinical studies on the anti-diabetic effects of extract of *M. charantia* should be encouraged. In addition, attention needs to be paid to the toxicity of *M. charantia* extract. Many toxicological studies have demonstrated in years past that extracts of *M. charantia* could be toxic in several organs of the body at varying doses. More recently a study on the reproductive toxicity of the plant in zebrafish confirm that it is teratogenic and cardiotoxic at certain dose [149]. Also, Abdillah and colleagues reported in 2020 that the administration of ethanolic extract of *M. charantia* for 28 consecutive days could have a toxic effect the liver and the kidney [150]. These reported toxic effects on vital organs of the body needs to be further elucidated so that a safe dose can be recommended for use [151].

## 8 Conclusion

The forgoing shows that *M. charantia* is a promising antidiabetic plant and could be of great use in the treatment of diabetes mellitus. Being, a phyto-substance, it is easily accessible and relatively cheap; hence, studies should be focused on the development of the plant into a widely acceptable anti-diabetic therapy, especially with a high level of global mortality accorded to diabetes mellitus amidst various anti-diabetic drugs coupled with the outrageous increase in the number of diabetic patients is foreseen.

## Declarations

### Author contribution statement

All authors listed have significantly contributed to the development and the writing of this article.

### Funding statement

This research did not receive any specific grant from funding agencies in the public, commercial, or not-for-profit sectors.

### Data availability statement

No data was used for the research described in the article.

### Declaration of interests statement

The authors declare no conflict of interest.

### Additional information

No additional information is available for this paper.
