# Corrigendum to “A detailed review on the phytochemical profiles and antidiabetic mechamisms of momordica charantia” [Heliyon 8 4 April 2022 Article e09253]

**DOI:** 10.1016/j.heliyon.2023.e22019

**Published:** 2023-11-02

**Authors:** Sunday Faith Oyelere, Oluwatobi Hezekiah Ajayi, Titilayo Eunice Ayoade, George Bueno Santana Pereira, Bolaji Charles Dayo Owoyemi, Ajibola Olaoluwa Ilesanmi, Olalekan Amos Akinyemi

**Affiliations:** a Institute of Biomedical Research, Universitat de Lleida, Spain; b Department of Biology, Georgia State University, USA; c Department of Physiology, Ladoke Akintola University of Technology, Ogbomoso, Nigeria; d Department of Chemistry, Federal University of Sao Carlos, Brazil; e Department of Biological Sciences, East Tennessee State University, USA; f Department of Toxicology and Cancer Biology, University of Kentucky, USA

The authors apologize for any potential confusion caused by the use of the phrase “glucose bigotry” in the original article.

The phrase was employed to characterize a condition wherein the body encounters difficulty in regulating blood sugar levels, typically associated with prediabetes or diabetes. This condition signifies an impediment in the effective processing of glucose (sugar), resulting in elevated blood sugar levels.

To improve the article the authors, correct the phrase “glucose bigotry” in line 2, section 3 to the well-recognized medical term “glucose intolerance.”

The authors would like to make apologies for any inconvenience caused.

